# Personality and the Moderating Effect of Mood on a Verbal Aggressiveness Risk Factor from Work Activities

**DOI:** 10.3390/jcm7120525

**Published:** 2018-12-07

**Authors:** María del Mar Molero Jurado, María del Carmen Pérez-Fuentes, Ana Belén Barragán Martín, María del Mar Simón Márquez, África Martos Martínez, José Jesús Gázquez Linares

**Affiliations:** 1 Department of Psychology, Faculty of Psychology, University of Almería, 04120 Almería, Spain; mmj130@ual.es (M.d.M.M.J.); abm410@ual.es (A.B.B.M.); msm112@ual.es (M.d.M.S.M.); amm521@ual.es (Á.M.M.); jlinares@ual.es (J.J.G.L.); 2 Department of Psychology, Universidad Autónoma de Chile, Región Metropolitana, Providencia 7500000, Chile

**Keywords:** personality, emotional aspects, communication, work activity

## Abstract

One of the trends in the current research in psychology is to explore how personal variables can determine a person’s communication style. Our objective was to find out the moderating effect of mood in the relationship between the five big personality traits and an aggressive verbal communication style risk factor from work activities in a sample of nursing professionals. This study is a quantitative descriptive design. The final sample was 596 nurses with an age range of 22 to 56 years. An ad hoc questionnaire was used to collect sociodemographic data, and the 10-item Big Five Inventory, the Communication Styles Inventory, and the Brief Emotional Intelligence Inventory for Senior Citizens were used. This study shows that, for nursing professionals, the agreeableness, conscientiousness, and neuroticism traits have a close relationship with aggressive verbal communication. Even though mood moderates this relationship, it is only significant for those individuals with high scores in neuroticism. Since personality dimensions are considered to be relatively stable over time and consistent from one situation to another, organizations should offer workshops and other types of practical activities to train workers in communication skills and emotional intelligence, in order to promote the health of employees and patients, and avoid risk factors from work activities in nursing.

## 1. Introduction

Communication is a basic function of human beings, of vital importance to developing interpersonal relationships, and for groups, organizations, and society to function well [1,2]. Since the 1970s, considerable academic and professional attention has been given to the study of communication styles, due to their practical relevance in any setting in which “transfer of personal information, knowledge, ideas, opinions and feelings play a fundamental role” [3] (p. 507). As a result of this scientific interest, the study of communication styles has increased in recent decades, with diverse lines of research emerging that have examined the phenomenon in different job contexts (e.g., education, organization, healthcare) [4,5,6,7]. Moreover, its importance in clinical and health contexts has been underlined in the literature. For example, effective communication between nursing professionals and their patients positively influences the health, satisfaction, and safety of patients [8,9,10].

The communication style concept was originally introduced by Norton [11] to refer to “the verbal and nonverbal interaction with signs which have literal meaning and must be understood, filtered and interpreted” (p. 99). Verbal aggressiveness [3], widely studied by Infante et al. [12,13], refers to a destructive communication style (taunts, threats, hostility, etc.) characterized by the use of hostile language, lacking in affect, and authoritarian, which does not facilitate dialogue, and can cause psychological damage to those who receive the message, in addition to negatively influencing the quality of interpersonal relationships [14].

One of the trends in current research in psychology is to explore how personal variables, such as personality, can determine a person’s communication style [2,15,16]. This influential line has developed based on the theoretical basis of the big five personality traits model (five-factor theory of personality) [17,18]. From this perspective, it is understood that individuals develop a certain communication style according to their personality traits, and the influence of social and cultural factors [7,19]. 

In the literature reviewed, low levels of agreeableness and conscientiousness, and high levels of neuroticism have been found to predict counterproductive behaviors in the workplace, specifically, the use of aggressive verbal language with coworkers and clients [20,21,22]. In a study by Grumm and von Collani [23], verbal aggressiveness was shown to be positively related to a personality profile characterized by high levels of neuroticism and low extraversion, agreeableness, conscientiousness, and openness to experience. Similarly, Barlett and Anderson [24] found the dimensions agreeableness, openness to experience, and neuroticism to be the best predictors of a wide range of violent behaviors, while authors such as Xie et al. [25] demonstrated that all personality traits—except neuroticism—could predict prosocial behavior. 

In addition to the above, some studies have explored the role of emotions with regard to aggressive behavior. One of the most-studied constructs is emotional intelligence (EI), referring to skills that people have for understanding, perceiving, and adaptively regulating their own emotions and those of others [26]. Some empirical studies have shown a significant relationship between low EI and aggressive verbal behavior [27,28]. 

Guo et al. [29] found that EI functions as a mediator between neuroticism and prosocial behavior. However, a relationship has also been found between the five personality traits and EI, especially with responsibility and neuroticism [30,31]. It has been suggested that EI is determinant for achieving personal and social success as well. Thus, people who manage their emotions adequately can cope with conflictive situations in an adaptive manner [32].

It has likewise been shown that positiveness and optimism (mood) [33] favor positive interpretation of potentially stressful situations, contributing to improving a person’s perception of being able to control their surroundings and, thereby, their wellbeing [34,35,36]. However, the relationship between positiveness and wellbeing is stronger in persons with high scores on extraversion, agreeableness, and conscientiousness [37]. 

Our objective was to find out the moderating effect of mood in the relationship between the five big personality traits and an aggressive verbal communication style in a sample of nursing professionals. 

## 2. Materials and Methods

### 2.1. Participants

The original sample was 619 nursing professionals, but 23 subjects were rejected (19 because random answers were detected by the control questions, and 4 because they had not completed the entire battery of questions), leaving a final sample of 596 nurses. 

The mean age of the participants was 31.53 years (Standard Deviation (*SD*) = 6.55) in a range of 22 to 56 years. The sex distribution in the sample was 83.7% (*n* = 499) women and 16.3% (*n* = 97) men, with a mean age of 31.56 (*SD* = 6.62) and 31.38 (*SD* = 6.21) years, respectively. The marital status of the participants was 53.7% (*n* = 320) single, 44.3% (*n* = 264) married or stable partner, 1.8% (*n* = 11) divorced or separated, and 0.2% (*n* = 1) widowed. Their employment situation at the time of the study was distributed as follows: 72.1% (*n* = 430) were working with a part-time contract and 27.9% (*n* = 166) with a stable contract. 

### 2.2. Instruments

An ad hoc questionnaire was used to collect sociodemographic data from the participants (age, sex, marital status) and, also, their current employment situation. 

The 10-item Big Five Inventory (BFI-10) [38] was applied for the personality dimensions. This is a brief version of the BI-44 scale [39,40], developed to provide a personality inventory for research with time limitations. It enables study of the five big personality factors (extraversion, conscientiousness, agreeableness, neuroticism, and openness to experience). Previous studies have demonstrated that the BFI-10 has psychometric properties comparable, in size and structure, to the complete BFI-10 scale. There are findings that back BFI-10 factor validity, construct validity, and criterion validity [38,41,42].

In addition, the Communication Styles Inventory [3] consists of 96 elements for evaluating communication behaviors. The items are divided into equal parts on 6 domain scales (16 items per scale): expressiveness, preciseness, verbal aggressiveness, questioningness, emotionality, and impression manipulativeness. Each domain scale has 4 facets, and each of these has 4 elements. The items are answered on a Likert-type scale, with answer choices from 1 (completely disagree) to 5 (completely agree). The authors reported Cronbach’s alpha on the scales varying from 0.82 to 0.88 in a sample from the general population, and 0.83 to 0.87 in a sample of students. In this case, we used the verbal aggressiveness scale with a Cronbach’s alpha of 0.78.

Finally, to measure mood, we used the scale with the same name included in the Brief Emotional Intelligence Inventory for Senior Citizens (EQ-i-20M) [43], validated and scaled by the authors for an adult Spanish population, adapted for adults from the Emotional Intelligence Inventory: Young Version (EQ-i-YV) by Bar-On and Parker [44]. It consists of 20 items with 4 answer choices arranged on a Likert-type scale. It is structured in 5 factors: intrapersonal, interpersonal, stress management, adaptability, and mood. The Cronbach’s alpha for the mood scale used in this study was α = 0.88.

### 2.3. Procedure

Before data collection, the participants were guaranteed compliance with information, confidentiality, and ethical standards in data processing. The study was approved by the Bioethics Committee of the University of Almería. The questionnaires were implemented on a web platform, which enabled the participants to fill them out online. A series of control questions were included to detect random or incongruent answers, which were then discarded from the study sample. 

### 2.4. Data Analysis

This study is a quantitative descriptive design. This paper also includes valuable recommendations for the revision of Strengthening the Reporting of Observational studies in Epidemiology (STROBE) [45]. First, frequency analyses were done to find out the distribution of the sample according to the sociodemographic variables, descriptive analyses, and Pearson’s correlation coefficient to identify the interactions between variables in the study. A stepwise multiple linear regression analysis was performed based on these data. SPSS v.23 (IBM, Armonk, NY, USA) [46] statistical software was used for these analyses. Then a simple moderation analysis was done to identify how mood moderates each of the dimensions of personality included in the regression analysis as predictors of verbal aggressiveness. The SPSS macro was used to compute simple moderation effect models [47]. Bootstrapping with 5000 bootstraps was used to estimate coefficients. 

## 3. Results

Table 1 shows the descriptive statistics and correlations between the study variables. A significant association was observed between verbal aggressiveness, and most of the personality factors. Specifically, there was a positive correlation with neuroticism (*r* = 0.30, *p* < 0.001) and a negative correlation with agreeableness (*r* = −0.35, *p* < 0.001), conscientiousness (*r* = −0.34, *p* < 0.001), and openness to experience (*r* = −0.13, *p* < 0.01). Mood correlated negatively with verbal aggressiveness (*r* = −0.40, *p* < 0.001).

The analysis of interactions between variables found correlations of mood with all of the personality factors: positive with extroversion (*r* = 0.08, *p* < 0.05), agreeableness (*r* = 0.23, *p* < 0.001), conscientiousness (*r* = 0.36, *p* < 0.001), and openness to experience (*r* = 0.23, *p* < 0.001), and negative with neuroticism (*r* = −0.40, *p* < 0.001).

### 3.1. Predictors of Verbal Aggressiveness in Nursing Personnel

As shown in Table 2, the regression analysis found four models, the last of which had the most explanatory capacity, with 28.2% (*R*^2^ = 0.28) of the variance explained by the factors included in the model (agreeableness, mood, conscientiousness, and neuroticism). 

To confirm the validity of the model, residual independence was analyzed. The Durbin–Watson (to evaluate if autocorrelation exists in a regression) D was 1.94, which confirms the absence of positive and negative self-correlation. Furthermore, it can be observed that the *t* was associated with a probability of error below 0.05 in all cases. The standardized coefficients reveal that the variable with the most explanatory weight was agreeableness, followed by mood. Finally, from the tolerance and variance inflation factor (VIF), the absence of collinearity among the variables included in the model may be assumed.

### 3.2. Moderating Effect of Mood on Predictive Value of Dimensions of Personality for Verbal Aggressiveness

According to Hair, Anderson, Tatham, and Black [48], moderating relationships entered could modify interpretation of the regression coefficients. The coefficients of the effects of each of the independent variables (agreeableness, conscientiousness, and neuroticism), the moderating variable (mood), and the interaction term on the dependent variable (verbal aggressiveness) were estimated based on simple moderation models. The figures below present the simple moderation models proposed for their analysis. 

The results of Model 1 report a statistically significant effect of mood (B_e_mood_ = −0.40, *p* < 0.01) and agreeableness (B_afab_ = −0.32, *p* < 0.01) on verbal aggressiveness. However, in this case, the coefficient of the interaction term is not significant (B_aree_ × _e_mood_ = 0.04, *p*= 0.30). Model 2, which takes conscientiousness as the independent factor, had similar results: a statistically significant effect on verbal aggressiveness, on both the independent variable (B_cons_ = −0.26, *p* < 0.05) and the variable considered a moderator (B_e_mood_ = −0.35, *p* < 0.05), but with no statistical significance of the interaction term coefficient (B_cons_ × _e_mood_ = 0.03, *p* = 0.38).

In Model 3, the effect of mood on verbal aggressiveness is statistically significant (B_e_mood_ = −0.47, *p* < 0.001), while the same is not true of the effect of neuroticism (B_neuro_ = −0.16, *p* = 0.10). However, in this case, the interaction term coefficient is significant (B_neuro_ × _e_mood_ = 0.08, *p* < 0.01), which shows that there is a moderation effect, where mood conditions the effect of neuroticism on verbal aggression. 

**Figure 1:**
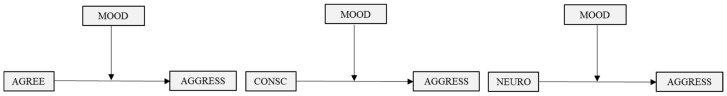
Simple moderation models proposed.

**Figure 2:**
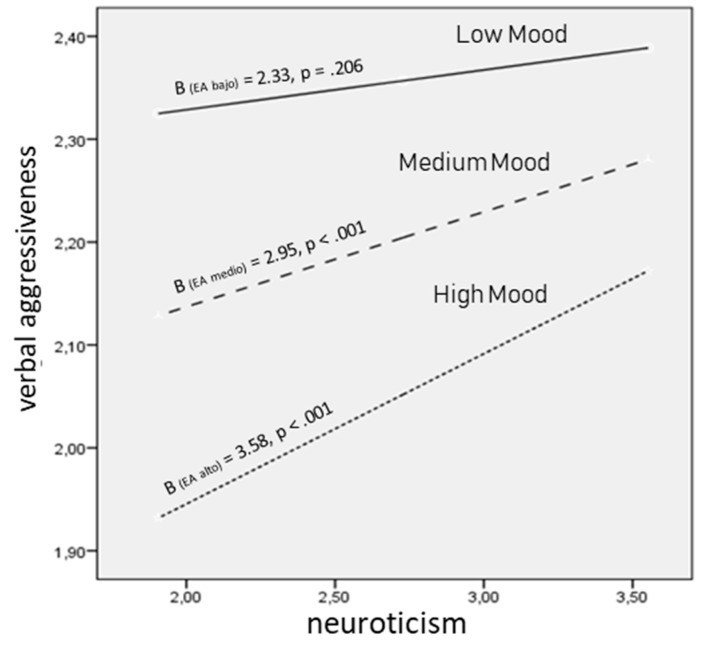
Interaction between neuroticism and mood in predicting verbal aggressiveness.

Then, using pick-a-point approach, the prediction of neuroticism on verbal aggressiveness was calculated for low, medium, and high mood. This shows the conditional effect of the independent variable on the dependent variable at different moderator strengths. Thus, the results shown in Figure 1 and Figure 2 suggest that the influence of the moderator variable comes about at medium (B = 2.95, *p* < 0.001) and high (B = 3.58, *p* < 0.001) mood. This implies that the moderating effect of mood takes place when it becomes medium to high.

Finally, the data found after application of the Johnson–Neyman technique make it possible to establish a wider range of moderator values and specify their involvement in the effect the independent variable exerts on the dependent variable. That is, when does the effect of the moderator begin to be significant? Specifically, when the mood score is greater than or equal to 2.50 (76% of the participants), neuroticism induces a stronger tendency toward verbal aggression. 

## 4. Discussion

This study shows that, for nursing professionals, the agreeableness, conscientiousness, and openness to experience factors maintain a significant negative relationship with the verbal aggressiveness communication style. On the contrary, it was found that the neuroticism trait has a close relationship with this disruptive style of communication, negatively affecting the nurse–patient relationship [8,9]. 

These results confirm what was previously found in other studies, suggesting that there is a close relationship between personality and verbal aggressiveness [20,22,23,25]. For instance, Bolton et al. [21] showed that workers with low levels of agreeableness and conscientiousness, and high levels of neuroticism, were more prone to use verbally aggressive language with their coworkers and clients. Similarly, Barlett and Anderson [24] demonstrated that agreeableness, openness to experience, and neuroticism were associated with a wide range of violent behavior. 

The data from our study also show a negative relationship between verbal aggressiveness and mood. These results confirm previous studies [28,32], which underlined the importance of adaptively regulating emotional information in social and work situations and avoiding aggressive behaviors. Along this line, it has been shown that positiveness and optimism are essential for interpreting potentially stressful situations more positively, especially in such an emotionally and psychologically challenging profession as nursing [34,35].

Moreover, our data also reveal a significant positive relationship between mood and all of the personality traits, except for the neuroticism dimension, with which it has a negative relationship. These results are consistent with previous studies, such as the meta-analysis by O’Boyle et al. [31], who found that EI had a significant positive relationship with extraversion, openness to experience, conscientiousness, and agreeableness, while it was the opposite with neuroticism. Joseph et al. [30] emphasized the relationship between conscientiousness, extraversion, and neuroticism with EI, as did Lui et al. [37], who demonstrated that mood predicted wellbeing in individuals with high scores in extraversion, agreeableness, and conscientiousness. 

According to our moderation analysis, neuroticism alone would not have a significant direct effect on verbal aggressiveness. However, it begins to be significant in interaction with mood. In fact, mood modulates the effect of personality on verbal aggressiveness more strongly as positiveness and happiness increase. These results agree with previous studies, in which it was proposed that personality traits partially determine communication styles [7]. Therefore, emotionally unstable persons faced with stressful situations tend to develop negative communication styles. However, only those with a positive attitude will be able to buffer the negative effects of their personality trait on the way they communicate [29].

This study has important practical implications for the job context. The relationship between personality traits and aggressive verbal communication must be emphasized, as well as the important effects EI has on this relationship. Since personality dimensions are considered relatively stable over time, and consistent from one situation to another [7], organizations should offer workshops and other types of practical activities to train workers in communication skills and EI, in order to promote the health of employees and patients. 

In like manner, the following limitations should also be considered. In the first place, the sample is made up of a majority of women, so the results may not be extendable to both genders. In the second place, the results cannot be generalized to the whole area of healthcare, because the sample used is very specific, so it would be recommended to enlarge the sample with other professionals. Finally, as the study design did not allow us to establish whether the relationships between the variables are stable over time, it would be interesting to carry out longitudinal studies to delve, more deeply, into the study of the influence of personality traits on communication style. It is a cross-sectional study, and its findings do not imply causality.

Future studies should widen the set of variables used, that is, include aspects related to the characteristics and working conditions (e.g., work areas, shifts, types of patient), in addition to considering all the facets of emotional intelligence and including other personal constructs, such as self-efficacy, for example, and performing a joint analysis of the interactions between them. 

## 5. Conclusions

In recent decades, there has been an exponential increase in scientific publications in which nursing professionals avoiding risk factors from work activities has been the subject of study. This interest derives from the characteristics and job contexts where they carry out their functions, as well as the important consequences and effects of their behavior on the wellbeing of patients and organizations. 

Our study was interested in evaluating the moderating effect of mood on the relationship between personality and verbal aggressiveness. The agreeableness, conscientiousness, and neuroticism traits have a close relationship with aggressive verbal communication. Even though mood moderates this relationship, it is only significant for those individuals with high scores in neuroticism.

## Figures and Tables

**Table 1:** Descriptive statistics and correlations.

	M	SD	1	2	3	4	5	6	7
Verbal aggressiveness	20.19	0.45							
Extraversion	30.29	0.81	−0.03						
Agreeableness	30.98	0.60	−0.35 ***	0.02					
Conscientiousness	30.71	0.66	−0.34 ***	0.15 ***	0.16 ***				
Neuroticism	20.73	0.82	0.30 ***	−0.10 **	−0.14 ***	−0.24 ***			
Openness to experience	30.48	0.75	−0.13 **	0.62 ***	0.16 ***	0.27 ***	−0.09 *		
Mood	20.96	0.62	−0.40 ***	0.08 *	0.23 ***	0.36 ***	−0.40 ***	0.23 ***	

Note: M = Means; SD = Standard Deviation; * The correlation is significant at 0.05; ** The correlation is significant at 0.01; *** The correlation is significant at 0.001.

**Table 2:** Stepwise multiple linear regression model for verbal aggressiveness.

**Model**	***R***	***R^2^***	**Corrected *R^2^***	**Change Statistics**				**Durbin−Watson**
				**Standard Error of Estimation**	**Change in *R^2^***	**Change in *F***	**Sig. of Change in *F***	
1	0.40	0.16	0.15	0.42	0.16	113.01	0.000	1.94
2	0.48	0.23	0.22	0.40	0.07	54.88	0.000	
3	0.51	0.26	0.26	0.39	0.03	29.35	0.000	
4	0.53	0.28	0.27	0.39	0.01	12.00	0.001	
**Model 4**		**Nonstandardized Coefficients**		**Standardized Coefficients**	***t***	***Sig.***	**Collinearity**	
		***B***	**Standard Error**	**Beta**			**Tol.**	**VIF**
(Constant)		3.70	0.16		22.06	0.000		
Mood		−0.15	0.03	−0.21	−5.35	0.000	0.74	1.35
Agreeableness		−0.19	0.02	−0.25	−6.96	0.000	0.93	1.06
Conscientiousness		−0.13	0.02	−0.19	−5.07	0.000	0.84	1.17
Neuroticism		0.07	0.02	0.13	3.46	0.001	0.82	1.21
